# Tolerance to Bone Marrow Transplantation: Do Mesenchymal Stromal Cells Still Have a Future for Acute or Chronic GvHD?

**DOI:** 10.3389/fimmu.2020.609063

**Published:** 2020-12-11

**Authors:** Martino Introna, Josée Golay

**Affiliations:** ^1^ Center of Cellular Therapy “G. Lanzani”, Division of Haematology, Azienda Socio-Sanitaria Territoriale Papa Giovanni XXIII, Bergamo, Italy; ^2^ Fondazione per la Ricerca Ospedale Maggiore, Bergamo, Italy

**Keywords:** mesenchymal stromal cell, graft versus host disease, hematopoietic stem cell transplantation, immunosuppressive drugs, inflammation

## Abstract

Mesenchymal Stromal Cells (MSCs) are fibroblast-like cells of mesodermal origin present in many tissues and which have the potential to differentiate to osteoblasts, adipocytes and chondroblasts. They also have a clear immunosuppressive and tissue regeneration potential. Indeed, the initial classification of MSCs as pluripotent stem cells, has turned into their identification as stromal progenitors. Due to the relatively simple procedures available to expand *in vitro* large numbers of GMP grade MSCs from a variety of different tissues, many clinical trials have tested their therapeutic potential *in vivo*. One pathological condition where MSCs have been quite extensively tested is steroid resistant (SR) graft versus host disease (GvHD), a devastating condition that may occur in acute or chronic form following allogeneic hematopoietic stem cell transplantation. The clinical and experimental results obtained have outlined a possible efficacy of MSCs, but unfortunately statistical significance in clinical studies has only rarely been reached and effects have been relatively limited in most cases. Nonetheless, the extremely complex pathogenetic mechanisms at the basis of GvHD, the fact that studies have been conducted often in patients who had been previously treated with multiple lines of therapy, the variable MSC doses and schedules administered in different trials, the lack of validated potency assays and clear biomarkers, the difference in MSC sources and production methods may have been major factors for this lack of clear efficacy *in vivo*. The heterogeneity of MSCs and their different stromal differentiation potential and biological activity may be better understood through more refined single cell sequencing and proteomic studies, where either an “anti-inflammatory” or a more “immunosuppressive” profile can be identified. We summarize the pathogenic mechanisms of acute and chronic GvHD and the role for MSCs. We suggest that systematic controlled clinical trials still need to be conducted in the most promising clinical settings, using better characterized cells and measuring efficacy with specific biomarkers, before strong conclusions can be drawn about the therapeutic potential of these cells in this context. The same analysis should be applied to other inflammatory, immune or degenerative diseases where MSCs may have a therapeutic potential.

## Introduction

The transplantation of hematopoietic stem cells (HSCT) from a normal donor to a “genetically matched” recipient is a current therapeutic option in onco-hematology. The most common toxicities of the procedure are rejection, disease relapse and acute and/or chronic graft versus host disease (aGvHD and cGvHD, respectively), even though these have been substantially reduced by the introduction of innovative transplantation procedures, wider donor availability with better donor selection, as well as the use of new drugs or new schedules of treatment and prophylaxis. The best example of an innovative prophylaxis treatment is the administration of high doses cyclophosphamide (Cy) post-transplant in order to promote tolerance, reviewed in (1, 2). Indeed, such treatment appears efficacious in reducing allo-reactive donor conventional T cells, while preserving the T regulatory compartment, possibly due to the high content in these cells of aldehyde dehydrogenase, an enzyme that favors chemoresistance (3).

In spite of the more recent reduction in the incidence rate of aGvHD and cGvHD during allogeneic HSCT, these conditions remain a difficult problem, since, in the most severe and resistant forms and after failure of steroid treatment, well defined and clearly effective second- or third-line treatments are not yet available. Fortunately this last statement may not be completely true anymore, since the JAK1/JAK2 inhibitor ruxolitinib has been rapidly approved by the FDA in October 2019 for the treatment of steroid refractory (SR) aGvHD, on the basis of significant results from a multi-center phase III trial (4) (and see below). Furthermore, this drug appears to show promising activity in cGvHD. Nonetheless, many other potential drugs are also being investigated for GvHD treatment, including Mesenchymal Stromal Cells (MSCs). We would therefore like to briefly summarize in this review the knowledge that has accumulated about the pathogenetic and immunological mechanisms behind acute and chronic GvHD (which underlie the lack of tolerance of the donor immune system to the host tissues, i.e. GvH tolerance), and concentrate our discussion specifically on the state of the art with regard to administration of human MSCs as a treatment strategy for such devastating diseases. We will treat the topics of aGvHD and cGvHD separately. The abbreviations used throughout the text are listed in **Table 1**.

**Table 1:** List of major abbreviations used in the text.

Abbreviation	Full name	Abbreviation	Full name
APC	Antigen presenting cell	LIF	Leukaemia inhibitory factor
ATG	Anti-thymocyte globulin	LN	Lymph node
BCR	B cell receptor	LPS	Lipopolysaccharide
BM	Bone marrow	NK	Natural killer cell
Breg	Regulatory B cell	NLRP	Nucleotide binding oligomerisation domain, leucine rich repeat and pyrin domain containing
CR	Complete response	MHC	Major histocompatibility complex
CsA	Cyclosporin A	MMF	Mycophenolate mofetil
Cy	Cyclophosphamide	MMPs	Matrix metalloproteases
DAMP	Damage associated molecular pattern	MSC	Mesenchymal stromal cell
DC	Dendritic cells	MTX	Methotrexate
ECP	Extracorporeal photopheresis	MØ	Macrophage
EV	extracellular vesicles	PAMP	Pathogen associated molecular pattern
FcγR	Fcgamma receptor	PGE2	Prostaglandin-E2
FDA	Federal Drug Agency (US)	PDGF	Platelet derived growth factor
GC	Germinal center	PR	Partial response
GM-CSF	Granulocyte-monocyte colony stimulating factor	ROS	Reactive oxygen species
GMP	Good manufacturing practice	SR	Steroid resistant
GvHD	Graft versus host disease (a: acute; c: chronic)	TCR	T cell receptor
HMGB1	High mobility group box 1	Tc	T cytotoxic (cytotoxic T cell)
HSCT	Hematopoietic stem cell transplantation	TGF	Tumour growth factor
IBMIR	Instant blood mediated inflammatory reaction	Th	T helper (helper T cell)
IDO	Indoleamine 2,3 dioxygenase	TIMPs	Tissue inhibitor of metalloprotease
ISCT	International Society of Cell Therapy	TLR	Toll like receptor
IFN	Interferon	TNF	Tumor necrosis factor
IL-	Interleukin-	Treg	Regulatory T cell
ISCT	International Society of Cell Therapy	Tr1	Regulatory Type 1 T cells

## The Biology and Immunology of aGvHD

Only to briefly recall the inflammatory context that underlines aGvHD, we will schematically summarize the main pathogenetic steps which take place in this condition.

The earliest pathophysiological event in the disease process (phase 1) is a diffuse endothelial damage, occurring as a consequence of the conditioning chemo-radiotherapy, which induces neo-angiogenesis as well as the infiltration of innate myeloid cells, neutrophils and monocytes into the intestinal tract. The release of superoxide radicals and other reactive oxygen species (ROS) by neutrophils is an essential physiological element of the innate immune response against invading pathogens. Inflammatory stimuli include sterile damage associated molecular pattern (DAMP) molecules (nucleic acids, intracellular proteins such as high mobility group box 1 (HMGB1), heat shock proteins, histones, actin, ATP and reactive oxygen species (ROS) and extracellular proteins such as hyaluronic acid and biglycan), alarmins released by cellular degranulation (constitutively expressed endogeneous molecules, e.g. IL-1α, IL-33), as well as inflammatory cytokines (e.g. IL-6, TNF). These promote the translocation across the impaired mucosal barrier to the underlying tissue layers of microbiota associated molecular pattern molecules (i.e. Pathogen Associated Molecular Pattern or PAMP, which include LPS, lipoproteins, peptidoglycans, flagellin, fungal components, viral nucleic acids). Bacterial colonization of the classical GvHD target organs, skin, and intestinal tract, as well as liver, has led to the hypothesis that bacterial transmigration is essential for the disease. Both DAMP and PAMP act on specific cellular receptors (5), PAMP being particularly engaged in activation of host antigen presenting cells (APCs) and subsequent priming of T cells to enhance alloantigen presentation. In both cases, Toll like receptors (TLR) pathways are triggered through receptors on the plasma membrane (TLR2, TLR4) and in endosomes (TLR3, TLR7/8, TLR9). TLR pathway activation induces IFNα production *via* transcriptional interferon response factors (IRFs). Particularly important, at this step, is the activation of the inflammasome multi-protein intracellular complexes, such as NLRP1 and NLRP3, which are able to rapidly activate the caspase family proteases, that generate the mature forms of IL-1β and IL-18 from inactive intracellular precursors and then release them into the extracellular milieu (during a process known as pyroptosis of monocytes, i.e. an inflammatory form of cell death) (6, 7). Pyroptosis is considered a mechanism to release DAMP molecules, such as IL-1α, HMGB1, and ATP (6). Activated cells secrete further cytokines, in particular TNF, IL-1α, IL-6, IL-33, IL-12, IL-23, type I IFN, and chemokines (e.g. CCL5), which enhance alloantigen presentation and expression of co-stimulatory molecules and cytokines by host APC. Host dendritic cells (DCs), inflammatory monocytes and neutrophils migrate from the damaged intestinal epithelium towards mesenteric lymph nodes, where donor T cells are activated. Moreover, IFNα and IFNγ can induce chemokines (CXCL9, CXCL10, and CXCL11) that recruit helper T cells 1 (Th1) and cytotoxic T cells 1 (Tc1) and NK cells, all expressing CXCR3 (5, 7–10).

During the second phase, allogeneic peptides presented by major histocompatibility complex (MHC) molecules are recognized by the T cell receptor (TCR) on conventional donor T cells (signal 1) in conjunction with many possible co-stimulatory molecules (CD40, OX40L, CD155/112, ICOSL) on recipient APCs (signal 2) which, together with cytokines such as IL-2, IL-12, IL-6, IL-23 (all signaling *via* JAK1/2)(signal 3), drive the differentiation of naïve T cells into mature helper and cytotoxic Th1/Tc1 and Th17/Tc17 effector cells (third phase). While the Th1/Th2 paradigm (Th1 being most important for aGvHD and Th2 for cGvHD) has been challenged and refined, the role of CD4 Th17 and CD8 Tc17 appears more relevant for both conditions and requires TGFβ/IL-6 and IL-1β/TNFα, respectively. Downstream effector cytokines (IL-2 and IFN-γ, secreted by Th1 and Tc1, respectively, and IL-17 produced by Th17 and Tc17, together with TNF and GM-CSF) cooperate with each other for the recruitment and activation of effector cells that induce target tissue apoptosis *via* FAS ligand and release of granzyme B and perforin (5, 7).

It has to be noted that, at the same time, the donor’s T cells may also be engaged in inhibitory interactions *via* other surface APC molecules such as CD86, CD80, Galectin 9, PDL-1/2 and, additionally, that the entire scenario is counterbalanced by the presence of the donor’s regulatory T cells: Tregs (CD4^+^CD25^high^ IL-2Rα^+^ FoxP3^+^ T cells, which require IL-2 for homeostatic proliferation) and Tr1, which bear inhibitory receptors such as TIGIT, CTLA-4, CD28, LAG3, ST2, produce inhibitory IL-10 and TGF-β cytokines and are activated mainly by IL-33, released by damaged cells *via* ST2, the IL-33 receptor. APCs also express inhibitory molecules that can down-modulate the immune response. Generally speaking, these “inhibitory” mechanisms can be viewed as the effort of the damaged tissue to repair and counteract the tissue damage, by inhibiting T cell responses and by the production and release of tissue repair factors such as keratinocyte growth factor (KGF) by fibroblasts, amphiregulin by Tregs, IL-22 by innate lymphoid cells type 3 and R spondin by fibroblasts (5, 7, 8).

**Figure 1:**
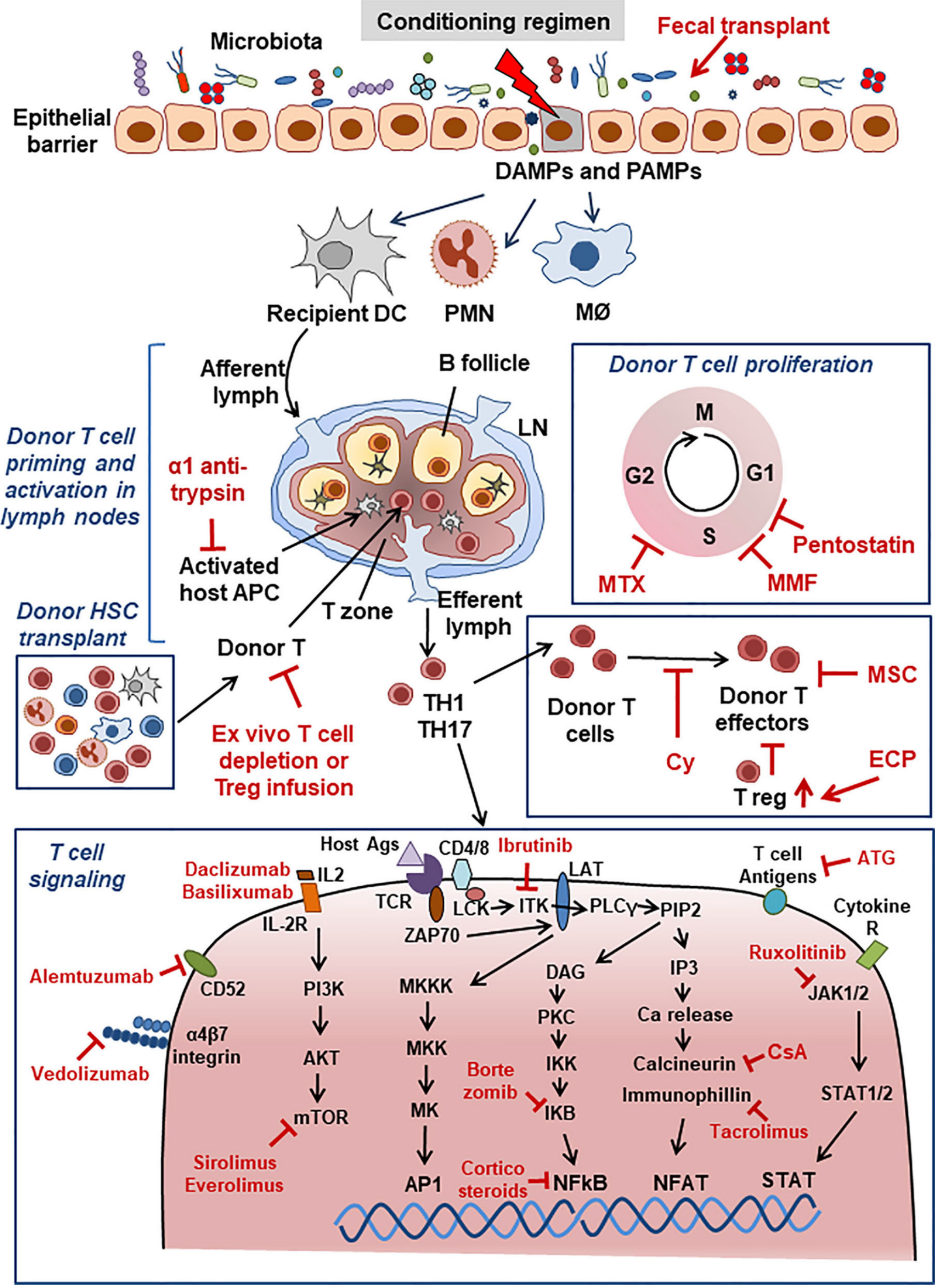
Acute GvHD. Schematic view of major aGvHD mechanisms and points of interaction with drugs used for aGvHD treatment. Drugs are shown in red font. For abbreviations see **Table 1**.

As is clear from the above summary, the immune activation and tissue damage that are involved in the triggering and establishment of aGvHD and cGvHD are complex and therefore offer a plethora of molecules/pathways that can be potentially modified by drug treatment. These elements are also the targets of drugs used to try and control GvHD in the clinic. **Figure 1** presents a very schematic and simplified view of the mechanisms of aGvHD induction.

## Treatment of aGvHD with Consolidated and Innovative Drugs

The recommended first-line treatment for aGvHD is systemic steroid therapy (aiming to inhibit immune cells activation and switch off the transcription of pro-inflammatory genes); however, about 35–50% of patients become refractory to steroid therapy. SR-aGvHD is generally defined as a clear progression after 3 to 5 days of treatment or no response after 5 to 7 days.

There has been up to very recently no accepted standard-of-care treatment for SR-aGvHD. This is due to the fact that in most cases clinical studies of SR-aGvHD are retrospective, single-arm, phase II studies, and cannot be easily compared with current patient populations due to the significant changes that have been introduced in recent years, not only in terms of supportive care, but also prophylaxis of aGvHD. Indeed this was a very recent conclusion made by the European Bone Marrow Transplantation GvHD management recommendation expert panel, which stated that “not enough data from well-designed studies are available to be able to compare the efficacies of the different second-line treatment options” (11). During the last several years, nonetheless, several drugs have been used as second-line therapy of SR-aGvHD, based empirically on the mechanisms of action described above and on the idea that blocking the donor’s T cells mediated attack on host tissues and associated acute inflammation would be beneficial and these are reported in **Table 2**. The most interesting is the already mentioned JAK1/2 inhibitor ruxolitinib, which has been approved in October 2019 by the FDA for the treatment of SR-aGvHD in adult and pediatric patients above 12 years old, based on the very recently published phase III clinical data showing an overall response rate at days 28 and 56 significantly higher in the 154 ruxolitinib treated patients compared to the 155 control group (4). Ruxolitinib finds a strong rationale in several aspects of the pathogenetic mechanism discussed above: it should be able to inhibit the activity of IFNγ, IL-6, IL-11, IL-12, IL-23, and IL-27 (which signal through JAK1/JAK2) and, possibly, also of IL-2, IL-4, IL-7, IL-9, IL-15, and IL-21 (which share JAK1 and JAK3 signaling molecules). Additionally, ruxolitinib has been shown *in vitro* to upregulate MHC-II expression and to block DC maturation, as well as inhibit neutrophilic migration, as discussed extensively elsewhere (12).

**Table 2:** Major drugs used as second line treatment for aGvHD and their mechanisms.

Drug^a^	Major mechanisms identified
Alemtuzumab	Humanised anti CD52 monoclonal antibody, lymphocytolitic
Alpha-1 antitrypsin	Inhibition of dendritic cells activation and induction of Tregs
Basiliximab, daclizumab	Monoclonal antibodies against CD25, IL-2 receptor alpha chain, inhibit T cells proliferation
Extracorporeal photopheresis (ECP)	Apoptosis and phagocytosis by APC leading to inhibition of pro-inflammatory cytokines, increased production of anti-inflammatory cytokines, induction of Tregs
Fecal microbiota transplant	Reconstitution of proper microbiota
Cellular therapy with MSCs	Multiple, in general “anti-inflammatory”/ immunosuppressive
Cellular therapy with T regs	Increase of circulating levels of Tregs
Ruxolitinib^b^	Inhibition of JAK1 and JAK2, major intracellular kinases mediating signalling of a variety of cytokines
Mycophenolate mofetil (MMF)	Blocks de novo pathway of purine synthesis in T lymphocytes, antiproliferative
Methotrexate (MTX)	Inhibition of nucleotides synthesis, block T cells proliferation
Pentostatin	Adenosine deaminase inhibitor, inhibits purine metabolism and blocks proliferation of T lymphocytes
Rabbit anti-thymocytes antibody (ATG)	Antibody against various T antigens, cytolytic for T lymphocytes
Sirolimus	Mammalian Target of Rapamycin (mTOR) inhibitor, blocks T cells activation
Vedolizumab	Monoclonal antibody anti α4β7 integrins, blocks gut homing of T lymphocytes

^a^These drugs are used as second line treatments for SR aGvHD, as reviewed by Penack et al. (11).

^b^Ruxolitinib has been recently approved by FDA as second line therapy for SR aGVHD.

Other treatments have also been commonly used to treat SR-sGvHD: Extracorporeal photopheresis (ECP), an immune-modulatory treatment able to induce apoptosis of T cells, anti-inflammatory and Th2-promoting cytokines and, as well as increase the levels of circulating Tregs; anti-thymocyte globulin (ATG) which induces not only T cell depletion, but also apoptosis of B cells, as well as upregulation of Tregs and NK cells; inhibitors of calcineurin (the TCR signaling intermediate to the NFAT transcription factor) such as tacrolimus and cyclosporine A (CsA) that inhibit TCR signaling; several monoclonal antibodies (mAbs) against IL-2Ra (daclizumab and basiliximab), IL-6R (tocilizumab), TNF receptor, or TNF-α (infliximab, etanercept); inhibitors of the downstream signaling mTOR molecule (sirolimus, everolimus), or dihydrofolate reductase inhibitors (methotrexate) which block production of thymidylate and purines and suppress T cell activation and proliferation.

Treatments that have fewer data available and are therefore considered to be third-line treatment options include alemtuzumab (anti–CD52 receptor antibody) which induces T cell and B cell depletion; pentostatin (a potent inhibitor of adenosine deaminase, the purine salvage enzyme involved in the irreversible deamination of adenosine and deoxyadenosine) and inhibitors of lymphocyte proliferation such as mycophenolate mofetil (MMF) (5, 13). Most recently, abatacept, a fusion protein that selectively inhibits T cell co-stimulation by binding to CD80/CD86 on APCs and blocking CD28-mediated signaling has been proposed (14).

Other recent proposed biological drugs or treatments have been introduced, whose development is based on the known pathogenesis of aGvHD, but are still in the early clinical phases of development. These include fecal microbiota transplant to re-establish the microbiota balance through infusion of a fecal suspension from a healthy donor into a patient’s gastro-intestinal tract, an anti CD3/CD7 immunotoxin to depletes T and NK cells, and finally vedolizumab, a mAb blocking the α4β7 integrin present on the surface of T lymphocytes and which inhibits their gastro-intestinal localization (5, 13). **Figure 1** summaries the major mechanisms of the drugs, shown in red, currently used for aGvHD treatment.

## Proposed Mechanism of Action of MSCs in aGvHD

Following the first report of the possible efficacy of MSCs in a case of SR-aGvHD (15)(see below), many biological studies have been pursued in an effort to better understand and ideally potentiate the immunosuppressive/anti-inflammatory mechanism of MSCs (16). Consequently, basic research has produced an impressive amount of data on the different mechanisms by which MSCs may have immunosuppressive activity in GvHD. These include the secretion of different immunosuppressive molecules, such as prostaglandins E2 (PGE2), indoleamine 2,3-dioxygenase (IDO), heme oxygenase-1, TGF-β, IL-10, nitric oxide, galectins 1, 3, and 9, Leukemia Inhibitory Factor (LIF) and HLA-G5, the stimulation and induction of Treg differentiation, the inhibition of Th17 differentiation, the induction of IL-10 production by CD5^+^ B cells (Bregs), the inhibition of B cells activation, proliferation and immunoglobulin secretion, as well as the inhibition of T and NK cell proliferation, the inhibition of IL-2 production by NK cells and the induction of T cells apoptosis. In addition, MSCs can dampen effector cell functions by cell-cell interactions *via* the PD-1/PDL-1 and HLA-G1 molecules. Furthermore, MSCs can secrete CCL2 and, through this chemokine, recruit monocytes and promote their differentiation to M2 type macrophages by upregulating expression of CD206, IL-10,and TGF-β and improve their phagocytic efficiency. MSCs can also inhibit monocyte differentiation into DCs and skew them into a more tolerogenic profile, reducing their expression of HLA-DR, CD1a, CD80, CD83, and IL-12 secretion. The monocytes/macrophages, after having phagocytosed MSCs, promote Foxp3^+^ Treg formation. Moreover, it has to be stressed that, once infused *in vivo*, and in general after reaching or being influenced by the milieu in inflammatory active conditions, MSCs receive most probably the necessary “licensing” or activating signals to acquire a full immunosuppressive anti-inflammatory profile (17). In particular IDO, IFN-γ, TNF-α, IL-1α, and IL-17, as well as TLR3 activation, are thought to enhance MSC-mediated immunosuppressive activity *in vivo*, which of course would be a positive effect of inflammation. The licensing phenomenon is evidenced by MHC class I and class II expression, increased ICAM-1 and VCAM-1 adhesion molecule expression, as well as IDO, IL-6, IL-8, hepatocyte growth factor (HGF), PGE2, PDL-1 and COX2 expression. The full activation of MSCs, that takes place in presence of both IFN-γ and TNF-α, induces expression of CCR10, CXCR3, CXCL9, and CXCL10 (18–24). All these possible mechanisms have been nicely reviewed recently, and therefore, we refer the reader to these works for greater details as well as summary figures and tables (18, 21, 23, 25, 26).

It should be stressed that MSCs, even in an allogeneic setting, are not themselves APCs because they lack expression of the co-stimulatory molecules CD80 and CD86, and of MHC class II antigens and show low expression of MHC class I molecules. Furthermore they probably quite rapidly disappear *in vivo* (enacting therefore a hit and run mechanism, see below) (24, 27). Thus, a plethora of molecules can participate to MSC mediated immunosuppression *in vivo*.

## Prophylaxis of aGvHD with MSCs

Prevention of GvHD (both acute and chronic) has been attempted by MSC infusion, generally given together with the HSC graft and in some cases with additional subsequent infusions up to 3 weeks following transplantation. These studies have been nicely reviewed recently by Morata-Tarifa et al., in a work which included the meta-analysis of 16 studies and a total of 654 patients (28). The data overall show a trend for a lower incidence of aGVHD, particularly grade IV and a reduced cGvHD, particularly extended cGvHD (see also below paragraph on cGvHD). No difference on overall survival between groups could however be identified in this prophylactic setting.

## Treatment of aGvHD with MSCs

MSCs have initially shown much promise in the setting of aGvHD treatment. Indeed, almost 20 years have elapsed since the first description of the treatment of a 9-year old boy, suffering from grade IV SR-aGvHD, using third party, bone marrow (BM) derived MSCs. The patient showed a complete response without any toxicity and a possible immunosuppressive role of MSCs was immediately hypothesized (15).

Following this report, a large number of phase I/II academic clinical trials have been conducted in severe SR (mainly acute) GvHD patients, treated with “similar” cells, derived from several different anatomical sources, expanded *in vitro* in various conditions and given with different schedules. A meta-analysis by Hashmi et al., including 13 non-randomized studies and comprising 336 patients, indicated a complete response rate (CR) of 28% with a 6 months survival rate of responders of 63%. Survival did not differ with respect to age, time of administration or dose of MSCs delivered (29). Similarly, a Cochrane-based extended meta-analysis of the outcome of treatment or prophylaxis with MSCs in acute or chronic GvHD, that included 12 randomized clinical trials and 879 patients, concluded that MSCs are not proven to be an effective therapy (30), despite the fact that a number of single reports suggest a positive effect of MSCs. Nonetheless, due to the considerable heterogeneity of the clinical results, and consistent, measurable, objective response in critical patients in most studies (22, 31), in the absence of clearly effective second- and third-line therapies, the use of cryopreserved unmatched allogeneic MSCs has become medical practice in many European countries. It was also originally recommended as a third line agent by the British Society of Blood and Marrow Transplantation (BSBMT) (22, 32), despite the fact that, more recently, the clinical commission report on GvHD treatment published by the UK National Health Service concluded that there was not enough evidence supporting the use of MSCs in GvHD patients (20). A more recent and complete review includes 14 clinical studies, reaching similar conclusions (20). Perhaps the most negative impact on the clinical arena were the results of the only placebo controlled phase III clinical trial, based on the infusion of BM derived MSCs (Remestemcel-L, produced by Mesoblast, although initially manufactured by Osiris Therapeutics under the name of Prochymal), which failed to meet its primary end-point (durable complete response lasting 28 days or more) either in 149 adults or 14 children (22, 33). However patients with liver involvement who received at least 1 cell infusion had a higher durable complete response and higher overall complete or partial response rate compared to the ones who received placebo administration (33). The patients were treated with 2 × 10^6^ MSCs/kg, twice weekly for 4 consecutive weeks (33). Furthermore, a single arm, prospective study which enrolled 241 children suggests some benefit of MSCs in children. These were treated with a median of 11 MSC infusions (2 × 10^6^/kg) following failure of conventional therapies; those with an early response to MSCs at day +28 appear to have also improved survival (34, 35). Nonetheless the Mesoblasts company’s Biological Licence Application for the treatment of pediatric SR- aGvHD with MSCs was rejected on October 2020 by the FDA, who recommended to conduct at least one additional randomized controlled trial in adults and/or children.

As general comments about the clinical trials of MSCs for aGvHD, one can say that the pathogenesis of this disease involves many molecules, cells and pathways, which vary also according to the anatomical site involved as well as time during disease development, as described briefly above. Just to make matters even more complex, the same molecules can in some cases play opposite pro- and anti-inflammatory roles according to the disease status: one canonical example is IL-33 whose administration in animal models of GvHD may result in attenuation or exacerbation of the disease, according to the schedule at which it is administered (8). Furthermore, most clinical trials have been performed on groups of patients who had seen 3 and up to 6 different lines of therapy before receiving MSCs. Thus, the fact that MSCs can interact with multiple molecules, cells and pathways renders the identification of the most appropriate time and administration route of MSCs as yet very difficult. Some other specific factors that have delayed the optimization of MSC use in GvHD are discussed in more details in the following paragraphs.

## Factors That Have Delayed the Optimization of Mesenchymal Stromal Cells Use in GvHD

### Heterogeneity of Cell Sources and Products

Cell therapy is naturally wrought in difficulties because of the potential variability of the products, linked to variable number of passages *in vitro*, heterogeneity of anatomical source (nowadays, MSC-like populations can be isolated from multiple tissues, including BM, adipose tissue, cord blood, umbilical cord wall and placenta, dental tissue, decidual endometrial blood as well as others), differences in composition due to their derivation from individual or pooled donors and the different culture conditions used (different media and additives, automated on non-automated methods). Furthermore, there is as yet no validated, standardized potency assay for the final drug product, as specifically underlined by the International Society of Cell Therapy MSC committee (36).

As an alternative to biological variability, a German group has expanded BM derived MSCs from 8 individual donors, pooled the cells at the moment of the first passage and then banked them at passage 2. Interestingly, the allo-suppressive potential of MSCs from individual donors was highly heterogeneous in mixed lymphocytes reactions *in vitro* (MLR), while the activity of the pooled MSC bank was reported to be significantly greater than the mean potency of the 8 individual donors. Indeed, the banked pooled MSCs demonstrated a reproducible and consistent allo-suppressive effect *in vitro* (37). This novel manufacturing protocol (referred to as “MSC Frankfurt am Main” or MSC FFM) was clinically tested in a first cohort of 51 children and 18 adults with refractory aGvHD (38) and, more recently, in a multicenter German trial, 92 patients have been treated, 88 with aGvHD grade III-IV. A median of three doses was administered without apparent toxicity, overall response rates were 82% and 81% at the first and last evaluation. At six months, the estimated overall survival was 64%, while the cumulative incidence of death from underlying disease was 3%, similarly favorable in children versus adults (39).

These data are encouraging that MSCs could be prepared in a more homogeneous and standardized way to offer perhaps more effective treatment. Interestingly the latter clinical use of MSCs was performed on the basis of the national hospital exemption authorization, which suggests also an innovative political strategy to cope with the national and international Good Manufacturing Practice (GMP) regulation which, in some cases, may delay testing of novel cell-based drugs in clinical trials (40–42).

### Heterogeneity of the Inflammatory Environment of aGvHD In Vivo and Lack of Predictive and Validated Markers

To complicate the matter further, there is a wide heterogeneity of the inflammatory environment in the recipient at the moment of the infusion, a very imprecise knowledge of the real *in vivo* mechanism of action of the cells in the different phases of the disease and in different tissues involved and a lack of predictive biomarkers. These drawbacks have been the subject of in-depth critical revisions and discussions to which we refer the reader (18, 20, 22, 43, 44).

Several markers of aGvHD activity or tissue damage had been initially identified (IL-2Rα, TNFR1, IL-8, hepatocyte growth factor (45), but these as well additional molecules or effector cells (such as Th1, Th17, CD4, CD8 cells, and IL-6, HLA-G), measured in clinical studies to predict or follow GvHD, have given rather inconsistent results (20). More robust data have been obtained by monitoring the antimicrobial Paneth cell protein regenerating islet derived protein 3A or Reg3A, as well as the IL-33 receptor ST2, leading to the definition of an algorithm called MAP taking into account both markers. This method has been recently validated in an international clinical study and shown to predict GvHD gravity, mortality and response to treatment (46), so that these markers have been developed as a commercial kit (47). Interestingly, both molecules derive from the gastrointestinal tract and have complementary roles in the pathophysiology of aGvHD (9). Paneth cells are retained at the intestinal crypt base and contain antimicrobial peptides, including defensins, lysozyme, phospholipase a2. Reg3A concentrates in the mucus of the internal part of the gut mucosa and physically separates the microbiota from intestinal cells. Activated APCs, damaged stromal, endothelial and epithelial cells, as well as T cells trigger the release of alarmins such as IL-33 that bind to its receptor, ST2 (9). The possible role of Reg3A and ST2 as *in vivo* markers for aGvHD is therefore promising but will need to be confirmed in larger studies.

### Difficulty in Tracing MSCs In Vivo and Unclear Pharmacodynamics

In addition to the problems mentioned above, it has proved difficult to detect infused MSCs *in vivo*. There is indeed a lack a solid evidence for their *in vivo* persistence (20). Since intravascular infusion is the most popular route for clinical MSC delivery, persistence of systemically infused MSCs has been mostly studied and these analyses have revealed that a large fraction of infused therapeutic cells are rapidly embolized and destroyed in the microvasculature after triggering an inflammatory reaction (23, 48, 49). Other reports suggest that infused MSCs trigger complement activation and that this results in their *in vivo* removal (48), and overall, very serious concerns have been recently raised on the hemocompatibility of the different MSC products to be injected. In synthesis, the most important potentially negative effects are linked with their highly procoagulant tissue factor (TF) activity, which is able to activate the instant blood mediated inflammatory reaction (IBMIR). There are several suggestions to perform preliminary test *in vitro* and *in vivo* on the products and it may also be useful to include therapeutics such as heparin to prevent the reaction (50). More in general the authors suggest to adopt a global safety strategy particularly for the MSCs derived from “alternative” sources, since the BM MSCs appear sufficiently safe, due to their extended clinical usage (50). As one recent example, careful *in vivo* toxicity study in both intra-arterially injected rats and intravenously treated mice with labeled human placenta derived decidual MSCs have been conducted and did not show any toxicity (51).

The observation that MSCs rapidly “disappear” *in vivo* has recently led to an alternative hypothesis as to their mechanism of action, which suggests that circulating MSCs may die by apoptosis, be engulfed by phagocytic cells and, in doing so, trigger IDO release and immunosuppression, as demonstrated in an experimental model (52). Indeed, further studies showed that patients displaying high *in vitro* cytotoxicity against MSCs, seemed to respond better to MSC therapy, while those with low or absent cytotoxic activity did not improve following MSC infusion, cytotoxicity thus possibly representing an innovative marker (52). Interestingly, this susceptibility to undergo apoptosis in a cytotoxic assay *in vitro* might also be used as a potency assay.

Regarding the rapid disappearance of the MSCs *in vivo*, alternative hypotheses have been proposed, in particular that only “fit” cells survive and reach the affected tissues. The observation that the therapeutic activity of freshly collected MSCs was clearly superior to frozen and thawed cells in a mouse colitis model supports this explanation (53).

In the same vein, consideration should be given also to the route of administration with respect to the persistence of the “drug” *in vivo*, an element which has certainly been underestimated in most clinical trials conducted so far: in a recent elegant experimental work, mouse colitis was successfully treated by intraperitoneal or subcutaneous, but not intravenous administration (53) and extracellular deposits have been associated with increased persistence of MSCs in both experimental (54) and clinical settings (55).

On the other hand, the therapeutic benefit of MSCs has also been attributed in large part to the so called “hit and run” mechanism mediated by the production of extracellular vesicles (EV) and the secretion of cytokines, chemokines and growth factors that exert their activities during the initial days following injection. MSCs can also exert their immunomodulatory effects on cells *via* direct cell-cell contact, in a paracrine fashion and *via* the release of soluble factors (see above). EV contain a large array of cellular modulatory proteins, messenger RNAs and microRNAs (miRNA). MSC-derived EV can inhibit T, B and NK cells, possibly *via* the shuttling of specific miRNAs into the target cells. The capacity of B cells and monocytes to engulf EV seems particularly strong and, in addition, uptake of EVs by monocytes leads to their differentiation toward an immunosuppressive M2 signature, able to enhance the function of regulatory T cells (21). MSCs can also exert their healing effects by transferring mitochondria to target cells. This appears to be an important mechanism to revert metabolic damage and prevent apoptosis in target cells (24, 56, 57).

Clearly much work is still needed to understand the mechanism of action of MSCs *in vivo* in the context of aGvHD and measure their efficacy.

## The Biology and Immunology of Chronic GvHD

Chronic GvHD remains a major cause of non-relapse mortality in patients who survive longer than 2 years after allogeneic HSCT, and negatively influences both quality of life and long-term outcome of this procedure. Indeed, the incidence and severity of cGvHD have increased over the last 10 years, despite the advent of novel treatments and improved clinical practice (58).

cGvHD can involve not only the epithelial target tissues affected in classic aGvHD (gastrointestinal tract, liver, skin, and lungs) but also any other organ system, including oral, esophageal, muscoloskeletal, joint, fascial, ocular, and lympho-hematopoietic systems, hair and nails, and genital tissues (59). Although the highly inflammatory state of cGvHD can manifest itself as polyserositis and polymyositis, chronic disease more often is characterized by fibrosis with little inflammation and involves one or multiple organs in the integumentary, muscoloskeletal, cardiovascular, respiratory, gastrointestinal, reproductive, and both central and peripheral nervous systems (59).

**Figure 2:**
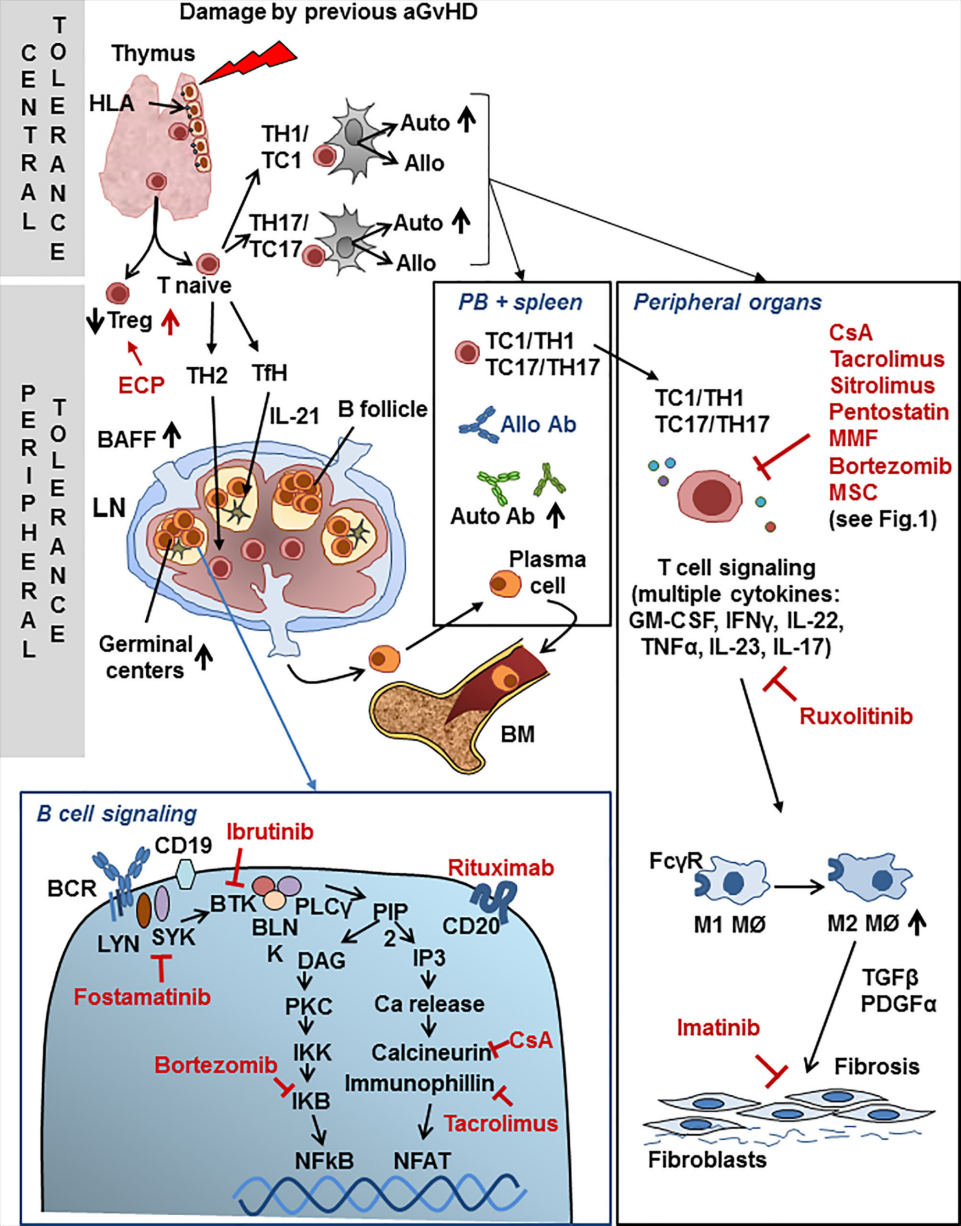
Chronic GvHD. Schematic view of major mechanisms specific to cGvHD and points of interaction with drugs used for second-line cGvHD treatment. Drugs are shown in red font. For abbreviations see **Table 1**.

Overall, cGvHD results from the excessive activation of immune effectors molecules and cells that cause inflammation in front of an insufficient presence of negative regulatory elements that help maintain tolerance (60). Schematically, the pathogenesis of cGvHD can be divided in 3 steps: 1) Early inflammation and tissue injury, both sustained by the innate immune system (not differently from aGvHD, see above). Endothelial cells contribute to the migration of donor’s T cells into secondary lymphoid organs, such as the spleen and lymph nodes (LNs) and subsequently into GvHD target tissues. DAMPs and PAMPs lead to increased antigen presentation by inflammatory monocytes, plasmacytoid and myeloid dendritic cells and B cells. 2) Adaptive activation of immune system effector cells leads to germinal center (GC) formation in cooperation with donor T follicular helper (Tfh) cells through soluble factors such as IL-21, while IL-17A is directly involved in monocyte-macrophage differentiation, driving the latter towards a pro-fibrotic phenotype (61). An important step is thymic injury, where medullary (mainly responsible for negative selection) and cortical thymic epithelial cells (responsible for positive selection) are targeted by alloreactive T cells, often during the previous acute phase of the disease (62), leading to subsequent loss of central tolerance and the development of donor-derived T cells with specificity for host target antigens. In addition, there is a general loss of regulatory cell populations, including Tregs, Bregs, NKregs, invariant NK/T cells (iNK/T) and regulatory type 1 T cells (Tr1), with consequent loss of peripheral tolerance. Besides an immune response against the host MHC proteins, T cell and B cell activation and antibody generation against neo-antigens can be observed. As an example, while high avidity interaction of B cell receptors (BCR) with auto-antigens in the BM normally results in deletion of auto-reactive B cells, this does not occur in cGvHD patients who develop antibodies to minor histocompatibility antigens. cGvHD is closely associated with abnormally high BAFF levels, an activated B cell phenotype and a high BAFF/B cells ratio. Excessive B-cell activation of the BCR and increased levels of soluble BAFF (sBAFF), an activation and survival factor for B cells, are thought to be the cause an altered BAFF:B cell ratio. Furthermore, the pathogenic B cells are resistant to apoptosis, contributing to increased cell survival and expansion in response to sBAFF of inappropriately selected auto- or alloantigen reactive cells. In any case, the BCR is strongly hyperactivated and so are the associated Syk and Bruton tyrosine kinase (BTK) signaling molecules (63). The reduced number of CD27^+^ memory and IgD negative post-GC B lymphocytes, together with increased infections, reduces the chances of a normal anti-microbial response. Why cGvHD patients produce allo-reactive B cells and antibodies, but do not show clinically relevant anti-microbial responses, still remains to be understood (60). 3) The propagation of tissue injury by dysregulated donor lymphocytes and aberrant tissue repair mechanisms set the stage for fibroblasts activation, collagen deposition, fibrosis and irreversible end-organ dysfunction, dominated by activated M2 macrophages that produce TGF-β and PDGF-α. Macrophages are major players in the control of inflammation, which has been shown to be an active, well defined process. A fundamental mechanism is the phagocytosis by macrophages of debris and of apoptotic neutrophils (efferocytosis). M1 type macrophages, exacerbate tissue damage and initiate the inflammatory response. In contrast, M2 type macrophages release ‘anti-inflammatory’ cytokines (e.g. IL-10, IL-1 receptor antagonist, IL-1RA, and the IL-1 type II decoy receptor), express high levels of scavenging receptors and specific chemokines and contribute significantly to the resolution of inflammation (64). It is worth noting at this point that this dichotomic clear-cut (M1/M2) separation is today considered an over-schematized view of an actually continuous plastic differentiation between functional macrophages attitudes (65). Following skin damage, efferocytosis and TGF-β may skew macrophage function (66). Activated Th2 and Th17 T cells promote fibrosis by secreting IL-13 and IL-17. The healing of damaged tissue must be coordinated together with the end of the inflammatory process. The current view is that the reparative mechanisms initiate while the inflammation induced by the alloreactive stimulus is controlled and this is then followed by the restoration of tolerance (60). B cell activation contributes with auto- (when donor immune response occurs against donor cells) and allo- (when donor cells respond to recipient cells) antibody production, and this further activates macrophages to release TGF-β. Indeed, macrophages express high levels of Fcgamma receptors (FcγRs) and efficiently bind and become activated by antibody coated (opsonized) targets, which in turn can generate very high levels of TGF-β (59, 60, 67). **Figure 2** summarizes the major mechanisms of cGvHD.

## Therapy of cGvHD

### Prophylactic Therapies Available for cGvHD Other Than MSCs

In most protocols for cGvHD prevention, anti-thymocyte globulin (ATG) is given in various combinations with methotrexate (MTX), cyclosporine A (CsA), tacrolimus, mycophenolate mofetil (MMF) or sirolimus before the HSC transplant. More recently post-transplant cyclophosphamide in various combination with most immunosuppressive drugs, has had a revolutionary impact on the prophylaxis of cGvHD (68). These drugs presumably act by depleting mostly T cells (ATG) and inhibiting activation of lymphocytes T and B cells.

### Standard Therapy for cGvHD

At present corticosteroids are the standard initial treatment of cGvHD, even though long-term steroid use results in infectious complications and other toxicities. Furthermore steroid resistance can occur. SR-cGvHD is defined as disease progression while on standard 1 mg/kg/day of prednisone for at least 2 weeks, stable disease at 4–8 weeks on 0.5 mg/kg/day or more of prednisone, or those unable to taper to less than 0.5 mg/kg/day.

In spite of the dramatic need for effective treatment and the enormous increase in our understanding of the pathogenetic mechanisms of cGvHD, consolidated second-line therapies are still lacking. As for the acute GvHD, also for the chronic form of GvHD the European Bone Marrow Transplantation GvHD management recommendation expert panel had reached the conclusion that “there are no data available allowing for comparison of the efficacy of different second-line treatment options for cGvHD and no standard second-line treatment exists” (see **Table 3**) (11). Nonetheless, a recent press release has announced that ruxolitinib has reached primary and key secondary endpoints in the phase III REACH3 trial comparing this Jak1/2 inhibitor to best available therapy, suggesting that this drug may become a standard second-line treatment also for cGvHD. Details of the results are therefore awaited. In the last few years, many exploratory clinical trials have been reported with the general perspective of reducing chronic inflammation and auto/allo B and T cell mediated immunity and results have been reported with a number of different drugs: rituximab which depletes B cells and therefore inhibits the allo-antibody response; ibrutinib, which irreversibly inhibits both Bruton tyrosine kinase (BTK) and IL-2 inducible tyrosine kinase (ITK), thus reducing B and T lymphocytes activation and additionally inhibits BAFF, IL-6, IL-4, and TNF-α production (69); fostamatinib, which specifically blocks the BCR associated SYK kinase; imatinib that inhibits TGFβ and PDGFRα signaling, and is therefore potentially active against fibrosis; ruxolitinib, a selective inhibitor of JAK1/2 (see above), low-dose subcutaneous IL-2, that induces an increase in Tregs; proteasome inhibitors (bortezomib), able to inhibit the degradation of IKB (NFKB inhibitor); extracorporeal photopheresis (ECP) with the aim of inducing the apoptosis of lymphocytes and facilitating the differentiation of DCs; others immunosuppressive agents previously reported for the treatment of the acute GvHD (calcineurin inhibitors, mycophenolate mofetil, mTOR inhibitors, pentostatin); finally, KD-025, an oral rho-associated coiled-coil kinase-2 (ROCK2) protein inhibitor is presently under investigation for the treatment of cGvHD (68). The most prevalent steps in the mechanism of action of cGvHD which are targeted by drugs are shown in **Figure 2**.

**Table 3:** Major drugs used as second line treatment of cGvHD and their mechanisms.

Drug^a^	Major mechanisms identified
Cyclosporin A, tacrolimus	Calcineurin inhibitors that block downstrem TCR signalling leading to NFAT regulated genes transcription; block T cells activation
Extracorporeal photopheresis (ECP)	Apoptosis and phagocytosis by APC leading to inhibition of pro-inflammatory cytokines, increased production of anti-inflammatory cytokines, induction of Tregs
Imatinib	Inhibits the abl kinase downstream of PDGFR and TGFβ receptors; inhibits fibroblasts proliferation and activation
Ibrutinib	BTK and ITK inhibitor: Inhibits B Cell Receptor (BCR) signalling and B cells activation and myeloid cell activation via inhibition of Bruton Tyrosine Kinase (BTK) expressed in B and myeloid cells
Sirolimus, everolimus	mTOR inhibitors that block T cells activation
Ruxolitinib	Inhibition of JAK1 and JAK2, major intracellular kinases mediating signalling of a variety of cytokines
Pentostatin	Adenosine deaminase inhibitor, inhibits purine metabolism and blocks proliferation of T lymphocytes
Rituximab	Monoclonal anti CD20 antibody, depletes B lymphocytes
Bortezomib	Proteasome inhibitor: Inhibits the proteolytic activity of proteasome in IkB degration, inhibits NFkB activation, inhibits T cells activation by cytokines
Fosfamatinib	Inhibits the BCR signalling via Spleen Tyrosine Kinase (SIK) inhibition in B lymphocytes and their activation
Mycophenolate mofetil (MMF)	Blocks de novo pathway of purine synthesis in T lymphocytes, antiproliferative

^a^These drugs are used as second line treatments for SR cGvHD, as reviewed in …by Penack et al. (11).

Given the variety of different organs affected during cGvHD, to a different extent in different patients, it is likely that the response to drugs may vary according to the disease site and that novel second-line therapies may not be as effective in all cases. Although new drugs have not provided a clear single option for all patients with cGvHD, promising complete response rates are starting to be observed. The general aim is of course to induce full tolerance while discontinuing immunosuppressive therapy, although it is presently still very difficult to identify those patients who will be responders and when immunosuppression can be tapered. In general, the standard immunosuppressive drugs are given until clinical amelioration and, later, slow tapering up to final discontinuation of drugs is the consolidated attitude. However, each attempt to taper drugs risks a subsequent return of GvHD and the need to restart immunosuppressive therapy at potentially higher dosages. Thus, currently, combination therapies incorporating novel drug targets and biomarkers are being investigated in clinical trials with the hope of diminishing toxicity while improving response rates (68).

## MSCs AS Prophylaxis for cGvHD

The report of Marata-Tarifa et al. mentioned above also includes a meta-analysis of 9 studies investigating the prophylactic use of MSCs for chronic GvHD prevention (28). The studies included 148 MSC treated patients and 236 controls, both adults and children. MSCs from BM or umbilical cord were given in most cases together with HSCs, with a second infusions at day +21 in one case. The analysis shows that MSC infusion was associated with reduced cGvHD incidence (RR = 0.64; 95% CI, 0.47–0.88, I^2^ = 0%).

The largest clinical study included in the meta-analysis described above is that of Gao et al. (70). This study directly addressed the issue as to whether prophylactic administration of umbilical cord derived MSCs was safe and could prevent cGvHD incidence in a multicenter, double blind, randomized controlled clinical trial in patients undergoing HLA haplo-identical HSCT (70). The MSC dose was a fixed monthly 3 × 10^7^ dose or saline as control, starting >4 months after transplantation in patients who had not developed aGvHD at day +100. 124 patients were enrolled (MSC N = 62, control n = 62). The average number of MSC infusions was 3.7 (range 2 to 4). cGvHD developed in 17 patients (24%): 14 mild/moderate, while 3 had a severe form. In the control group, cGvHD occurred in 30 patients (48,4%): 22 mild/moderate and 8 severe (p<0.05). No acute infusional toxicity nor adverse event were reported. 41 patients in the MSC group and 38 in the control group were still alive at the median follow up of 51 months (range, 24–70). Overall T cells numbers did not change, although Treg counts and the Th1/Th2 ratio increased after MSC infusion (p<0.05). Furthermore, the absolute numbers of memory B and NK cells in the MSC treated patient group were increased (70).

These data suggest that MSCs may have activity in cGvHD and larger controlled studies that include carefully studied biomarkers are warranted.

## Use of MSCs for cGVHD Treatment

The first clinical study of MSCs for the treatment of cGvHD reports the results of 19 patients treated with a median dose of 0.6 × 10^6^/kg of third party BM-derived MSCs, for one (n = 8) up to five doses (n = 1) (71). CR (n = 4) or PR (n = 10) were reported for a total 14 responders (73.7%). Immunosuppression was tapered after median 697 days in 5/14 survivors. Five patients (26.3%) died after the first MSC infusion. Reasons for death were invasive fungal infection (n = 2), primary malignant disease relapse (n = 2) and bronchiolitis obliterans (n = 1), this latter cause being related to cGVHD. No adverse events induced by MSCs were noted. The 2-year survival rate was 77.7%. MSCs seemed to be more effective for patients with cGVHD of the gastrointestinal tract or with liver and skin involvement. Interestingly, two patients with severe scleroderma had a PR or a minor PR and, clinical symptoms improved in patients with keratoconjunctivitis sicca. Clinical improvement was accompanied by an increased ratio of CD5^+^CD19^+^/CD5^−^CD19^+^ B and CD8^+^CD28^−^/CD8^+^CD28^+^ T lymphocytes suggesting effects on the immune system were taking place (71).

In a second study, four sclerodermic cGvHD patients were treated (72). The patients received four to eight infusions of 1 to 2 × 10^7^ third-party donor BM-derived MSCs intra-bone. All four patients showed an improved clinical score and a reduction of symptoms (mainly sclerodermic), with a 14.1-month median follow-up and standard immunosuppressive drugs could be tapered to a significant extent. From the laboratory investigations, it was clear that the proportion of IL-10- and IL-4-producing cells gradually decreased, whereas the proportion of IL-2- and IFNγ-producing cells increased consistently in all patients (72).

In a third report, a total of 23 refractory cGvHD patients received three infusions of third party BM derived MSC at 10^6^ cells/kg per infusion at 4 weeks intervals (73). 20/23 patients demonstrated an overall CR or PR at 12 months. Two PR patients died of fungal pneumonia, and three CR/PR patients died of leukemia relapse. Interestingly, best responses were observed in 16/23 with skin symptoms, 13/18 with oral mucosa and 13/15 with liver involvement. In most of the patients who achieved either CR or PR, the best therapeutic effects were observed 3 months after the first MSC infusion. In the responders, the absolute numbers of Bregs increased (74, 75). This was put in relation with *in vitro* data showing a higher survival rate and proliferation of CD5^+^ B cells and an increased frequency of CD5^+^IL-10^+^ Bregs after co-culture with MSCs. The data presented finally suggested that MSCs can induce Breg *via* IDO and that this may be an immunosuppressive mechanism in cGvHD (73).

A more recent article reported the results obtained with 11 patients with severe, refractory, cGvHD treated with repeated infusions of allogeneic BM-derived MSCs over 6 to 12 months period, i.e. an extended schedule (76). At least 6 doses of 2 × 10^6^ MSC/kg were administered with additional one to three doses to responders, while patients with progressive disease were taken off the study. Importantly, no patient could be defined as a non-responder until at least 6 months of treatment had been administered. With a median follow-up time of 76 months (range, 34–99) from inclusion, two patients have discontinued all systemic immunosuppression, and two have reduced steroids and calcineurin inhibitor. Organ responses were seen in joints (n = 8), skin (n = 4), eyes (n = 4), mouth (n = 3), gastrointestinal tract, and liver (n = 1 each). MSC treatment was well tolerated without immediate side effects. Overall, 6/11 patients showed long-term PR. 5 patients suffered grade 3 infections and 2 had dysplasia, as severe adverse events. The clinical effects were paralleled by reduced inflammatory cytokine levels and skin histology in the responders. Interestingly, the absolute number of naïve, but not memory T-cells, as well as the absolute numbers of naïve B cells (CD19^+^IgD^+^CD38low) and the CD31^+^ CD4 subpopulation (early thymus exiters) at the time of treatment were higher in responders compared to non-responders. Finally, CXCL10, CXCL2 and CCL2 levels (mainly produced by inflammatory monocytes) decreased during treatment in long-term responders, while they were upregulated in non-responders, suggesting a continuation or worsening of the inflammation in the latter patients.

These results are important and suggest that the immune status of patients even before MSC infusion may influence treatment response, which may allow to predict which patients will benefit from MSC treatment. Furthermore the study identifies biomarkers that correlate with response during time (76). Clearly a larger study will need to confirm these results.

In good agreement with the pathogenetic mechanisms of cGvHD discussed above, it is interesting to observe that independent biochemical spectrometric studies have identified several molecules as likely candidates to become cGvHD markers, such as CXCL9, CXCL10, ST2, MMP-3, osteopontin, BAFF, the macrophage scavenging receptor CD163 and DKK3 (Dickkopf-related protein 3), as recently reviewed (77).

A conclusion that seems to emerge from the analysis of all clinical trials of MSCs in the treatment or prophylaxis of cGvHD described above is that MSCs may indeed have beneficial clinical activity in this setting. It is probably important to administer repeated doses of cells to obtain a significant effect. Clearly larger controlled trials, investigating specific biomarkers of response are therefore necessary, as well as a careful long-term clinical evaluation of the chronic lesions and of the possibility of tapering the standard immunosuppressive regimens. Finally, it seems a good suggestion to try and “equalise” a standard pool of MSCs obtained from several donors, which could also easily benefit from recently introduced “closed” standardized bioreactors in order to generate more homogeneous MSC preparations for therapeutic purposes (78).

## One General Comment on “Tolerance” in the Context of GvHD

An extensive transcriptional profiling and statistical analysis of peripheral blood mononuclear cells from HSCT recipients has shown recently that, upon discontinuation of immunosuppressive therapy, two group of patients could be distinguished (from both acute and chronic GvHD): the ones who did not need any more drug therapy (tolerant) and the ones who still needed it (non-tolerant). The analysis of the identified genes confirmed the immunological nature GvHD and suggested a major role for NK cells, antigen presentation, lymphocyte proliferation and apoptosis (79).

Moreover, in a more recent updated analysis, the same group of researchers re-evaluated the consequence of immune suppression discontinuation on the HSC recipients. The results suggest that, during HSCT with standard immunosuppressive drugs and myeloablative conditioning, patients do not rapidly reach tolerance and tapering immune suppression therapy early does not prevent cancer relapse. Indeed, only 20% of patients were immune suppression-free survivors 5 years after HSCT. Interestingly, when all the variables associated with a successful discontinuation of therapy were analyzed, only the peripheral blood stem cells emerged to be significantly associated with an adverse event, in case of discontinuation of immunosuppression, suggesting, one more time, that the whole BM explants may offer an advantage over apheretic material (perhaps due to the well-known presence of MSCs in this tissue) (80).

These above interesting studies need obviously to be confirmed and extended.

## The Origin and Diversity of Mesenchymal Stromal Cells

The first demonstration of the presence in BM and other hematopoietic tissues of clonogenic progenitor cells capable of differentiating to fibroblasts as well as other mesodermal cells was published in the 1960’s by Alexander Friedenstein (81). Haynesworth later set up the culture system to expand BM MSCs in the early 1990’ [reviewed in (24)].

The notion of the stemness of MSCs was a concept initially proposed by Friedenstein (81, 82). Indeed, in the first 20 years following their initial characterization, there has been a diffuse emphasis on the stemness and pluripotency of MSCs, with a suggested unlimited differentiation capacity of these cells, indicating that they may be multipotent adult progenitor cells (83, 84). However already in 2005, the International Society of Cell Therapy (ISCT) published a position statement in Cytotherapy (85) clarifying the recommended designation of the cells in Multipotent Mesenchymal Stromal Cells (MSC) rather than Stem Cells, suggesting to abandon the stemness concept. In a subsequent position statement by ISCT, minimal criteria to define MSCs included the ability to differentiate to osteoblasts, adipocytes and chondroblasts, thus underlying their trilineage mesenchymal differentiation capacity, in addition to the property of adhesion to plastic surface and expression of CD73, CD90, CD105, but not CD34, CD45, CD14/CD11b, CD79a/CD19 and HLA-DR (immune lineage negative) (86). The subsequent experimental work, up to the present day, has indeed enormously revised this concept of the stemness of MSCs and, rather, their *in vitro* capacity to differentiate towards adipocytes, osteoblasts and cartilage, led to the redefinition of their property as stromal progenitor cells. Nowadays, the term “stromalness” is accepted as more appropriate than stemness for MSCs from different anatomical sources (44, 87). This definition does not rule out, obviously, that the ex-vivo expansion may hinder the presence of minimal subpopulations of true stem cells among the primary tissue MSCs, that may change their differentiation potential during ex vivo manipulations (see below).

MSCs are hypothesized to be present in tissues in the form of CD146^+^ pericytes and adventitial cells in the perivascular niche, as well as interstitial fibroblast-like cells in most organs and tissues. It has been shown however that the transcriptome of these cells changes significantly during *in vitro* culture and expansion, so that it is unclear whether the biological properties of MSCs *in vitro* really reflect those of their tissue progenitors (24). This question is of interest but obviously quite difficult to unravel.

“MSCs” are characterized by rather non-specific markers which do not allow to distinguish MSCs from different sources and with different biological properties. Furthermore work performed in the last 10 years has clarified that CD34^-^CD45^-^CD146^+^ “MSCs” isolated from different tissues have epigenetically different transcriptomes and differentiation programmes which are consistent with the tissue from which they have been isolated. Thus BM 146^+^ cells are capable of giving rise to bone and BM stroma that support hematopoiesis such as adipocytes, but are not myogenic or chondrogenic *in vivo*. Muscle-derived CD146^+^ cells are not skeletogenic and are myogenic, and cord blood-derived CD146^+^ cells are not myogenic but are chondro-osteoprogenitors and able to form cartilage *in vivo*. Thus the selective purification of CD34^-^CD45^-^CD146^+^ cells from several organs, leads to isolation of committed tissue-specific progenitors, not of multipotent or stem cells (88).

In the same vein, despite the standard expansion protocols generally applied for the production of clinical grade MSCs, differences have emerged upon careful analysis of genome wide methylation status, immunophenotype, transcription pattern and *in vivo* properties. These studies have shown for example that BM-derived MSCs spontaneously form a BM cavity in NOD SCID Gamma (NSG) mice *in vivo* through a vascularized cartilage intermediate that is progressively replaced by hematopoietic tissue or bone, at variance with MSCs derived from all other different sources, mainly adipose tissue, umbilical cord and skin (89). These observations may suggest a latent epigenetic program for endochondral differentiation present in BM-MSC and support the observation that BM-MSCs robustly build a functional human marrow niche whereas other MSCs may not (89).

Moreover, the important theme of MSC heterogeneity has been recently and extensively reviewed, with aspects such as donor variability, isolation procedures ex vivo, as well as differences in anatomical source being discussed. For example, a quite different proliferation potential has been observed *in vitro* in different MSCs with widely different timing for the appearance of senescence markers: senescence markers appeared already at passage 7 in BM derived MSCs, whereas adipose tissue derived, umbilical cord derived or endometrial derived MSCs showed much slower appearance of these markers (8, over 16 and 25–30 passages without sign of senescence, respectively) (44).

Similarly, heterogeneity is emerging in both xenogeneic and humanized mouse models of GvHD using adipose tissue, umbilical cord and BM derived MSCs, suggesting possible higher efficacy of umbilical cord and BM derived MSCs [carefully and extensively reviewed in (90)]. Nonetheless, overall, different MSCs administered in various dosages and schedules have shown only occasionally a statistically significant therapeutic effect. Whether this is due to inadequate models, MSCs sources or dosages is still unclear (90). The question of the MSC sources and specific biological and therapeutic properties therefore still needs to be better understood in the context of GvHD.

## MSCs in Tissue Repair and Fibrosis

It is worth pointing out at this stage that, whereas MSCs, as described above, may act as anti-inflammatory and immunosuppressive elements allowing the restoration of some tolerance, they may also play a role as a differentiation inducing and regenerative therapy, and this may play a role in the context of the chronic tissue damage seen in cGvHD.

This alternative “regenerative” role played by MSCs, largely explored in experimental and clinical settings of chronic lesions, might be reconducted to the secretion and paracrine effect of many molecules, such as vascular Endothelial Growth Factor, Fibroblast Growth Factor, Hepatocyte Growth Factor, Placental Growth Factor, Monocyte Chemoattractant Protein 1 (CCL2), Stromal differentiation factor-1, Ang-1, all critical for vascularization, as well as BCL-2, survivin, Insulin Growth Factor-I, Stanniocalcin-1 (STC-1), TGF-β, GM-CSF, all factors that inhibit cellular apoptosis and restore tissue homeostasis (23). Similar to the immune regulatory role of MSCs, their tissue repair capacity is thought by most authors to be mediated by the release either of EV or by intracellular components of the MSCs rapidly dying *in vivo* (48 h in most experimental animal studies, a hit and run mechanism as described previously).

Nonetheless, the most perplexing observation derives from the effects of MSCs on fibrosis mainly described in chronic inflammation (as for the cGvHD). MSCs have been shown in different contexts to ameliorate fibrosis by reducing the extent of monocyte/macrophage and B lymphocyte infiltration and inhibiting the expression of pro-inflammatory cytokines such as TNFα and IL-1β in liver and pulmonary fibrosis. As already mentioned above, MSCs appear able to reprogram pro-inflammatory macrophages (M1) towards an anti-inflammatory phenotype (M2), resulting in resolution of inflammation [as one example, see (91)]. In other experimental models, BM MSC have shown the ability to reduce liver fibrosis *via* induction of expression of MMPs by macrophages. Interestingly, also MSC-derived exosomes (see below) are able to reduce pulmonary fibrosis. To understand the role and mechanism of MSCs, it is worth noting that fibrosis appears to be the result of complex multiple interactions between molecules able to positively induce collagen deposition, as exemplified by TGFβ (see below), which appears also to be able to directly regulate the equilibrium between MMPs and tissue inhibitors of metalloproteinases (TIMPs). Also Wnt, one of the major signaling pathways involved in collagen deposition, is negatively regulated by Dickkopf protein 1 (Dkk-1), which is also under TGFβ control. In addition, as discussed above, MSCs are certainly able to induce Tregs, which are known to inhibit fibrocyte recruitment and fibrosis. Thus the *in vivo* anti-fibrotic activity of MSCs in liver, pulmonary and renal fibrosis is complex and, even if the local mechanism involved is far from being clarified, it has been very accurately detailed in a recent review article by Rockel et al. (92).

In contrast, MSCs may “contribute” directly to fibrosis. Indeed, the cell type most involved in development of fibrosis is the myofibroblast, which can secrete extracellular matrix proteins such as collagen and fibronectin. Many studies have demonstrated the plasticity of such cells as well as their organ specific developmental origin and localization. The pericytes, as well as fibroblasts and circulating BM derived mesodermic progenitors are believed to give rise to myofibroblasts and thus contribute to pathogenic fibrosis. Therefore, there is an emerging overlapping (from a morphological, phenotypic and *in vitro* differentiating potential point of view) between the cells which appear to be the MSCs precursors (pericytes) and the cells which appear to be the responsible of local fibrosis, even if a complete understanding and definition of such developmental programmes is far from being clarified. Nonetheless, it is tempting to speculate that what we define as *in vitro* expanded MSCs may have common properties and origin with the MSC-like cells which are responsible for tissue fibrosis and organ damage. The overall picture is also complicated by the observed plasticity *in vivo* in several experimental models which lead to the refusal of the concept of myofibroblasts as terminally differentiated cells, but rather as a transitory state in continuous evolution between deposition and regression of fibrosis (93).

Thus, the role of MSCs in the development and resolution of fibrotic conditions in cGvHD needs to be more fully understood to better explore how to manipulate these cells for their best therapeutic effects.

## MSCs Beyond GvHD: From the “Universal Drug for any Disease” to More Solid Clinical Perspectives

MSCs have been proposed and tested in many different clinical conditions beyond GvHD. Indeed the original misinterpretation on the stemness nature of MSCs and hypothesized multi-lineage differentiation, has unfortunately been abused by some private “direct to consumers” clinics, which have marketed MSCs to treat patients affected by a wide range of diseases and pathological conditions, without a strong rationale or scientific link between these cells and the disease etio-pathogenesis, with little subsequent demonstrated benefit and in some cases even resulting in adverse reactions. This critical point has been already raised by detailed position papers (49, 94–96).

Interestingly, in early 2019, a search of the “clinicaltrial.gov” website, using as keywords “MSC”, “mesenchymal cells” “stromal cells”, evidenced the registration of over 900 clinical studies globally (44), including more than 10.000 patients treated and ten phase III studies (97). In addition to Prochymal remestemcell, as discussed before, the use of MSCs was approved in Japan following the act on the safety of regenerative medicine (98). In 2018 the European Medicines Agency has approved Alofisel to treat Crohn’s disease (99). A search performed in april 2020 identified 12 different proprietary allogeneic and 4 autologous MSC products, utilized in 1094 ongoing clinical trials, 64 being phase II/III and 6 phase III/IV studies (including 47 studies for GvHD) (18). Approval was granted for allogeneic MSC therapies in Europe, Japan and India, such as Alofisel for the treatment of perianal fistulas in Crohn’s patients, based on the results of the Adipose Derived Mesenchymal Stem Cells for Induction of Remission in Perianal Fistulizing Crohn’s Disease (ADMIRE-CD) phase III study. Mesoblast’s TEMCELL HS was approved in Japan for the treatment of aGvHD in BM transplant recipients. Stempeucel, marketed by Stempeutics, has received limited approval in India for the treatment of critical limb ischemia (associated with Buerger’s disease) (100). In another review, Godoy and co-authors report 16 MSC-based commercial products, 6 for bone regeneration, 2 for perianal fistulas, 2 for regeneration of subcutaneous tissue, 1 for wound repair, 1 for cartilage repair, 1 for traumatic osteoarthritis, 1 for GvHD, 1 for acute myocardial infarction, 1 for acute radiation injury (18).

It is reassuring, therefore, that these approvals so far concern pathological conditions which can be reconciled with the mesodermic “stromalness” previously described (conjugated with anti-inflammatory and immune-suppressive activity). Indeed these activities are widely justifiable in diseases such as GvHDs and Crohn’s, due to the autoimmune and inflammatory pathogenesis of most connective tissue diseases. In contrast, the very unrealistic and unlikely activities in the direction of the pluripotent stemness are disappearing.

Nonetheless, the increasing understanding of the true heterogeneity of the MSC preparations obtained from different sources and by different methods, may in the future lead to a more precise identification of ideal tissue targeting for MSC products. Whereas the immunosuppressive and anti-inflammatory capacity of different MSC preparations may differ, as described above, this argument has not yet been addressed with respect to their use in different clinical conditions. For example, some clinical uses of MSCs (closing fistulae or wound repair), may require a “fibroblastic” ability, whereas others (treating the sclerotic lesions in cGvHD) will not. Rather, in the latter case, the immunosuppressive and anti-inflammatory activities shown by MSCs may rather induce the de-differentiation of myofibroblasts.

Lastly, but not less important, is the consideration that, in trying to cope with the clinical demand, several “GMP compliant” expansion methods ex vivo are available and have been applied, which may, by themselves, represent a confounding factor, as for the different anatomical source, individual donor variability, isolation procedures, and expansion conditions, as well as final formulation, scaling up, dosages, release tests and routes of administration. Many of these issues have been recently reviewed (44). As one example, crucial aspects of the supposed *in vivo* activity of MSCs, such as the immunosuppression of T and B cells, have been found to differ according to the different GMP compliant expansion protocols used (101, 102). Ideally, disease specific MSCs will soon be identified, in conjunction with optimized culture conditions and manufacturing.

## Conclusions

Despite several decades of *in vitro* and *in vivo* studies on MSCs, their ready availability from different tissues and their multiple functions have led to the conduction of many clinical trials and the approval of several commercial products for different clinical conditions, including GvHD. MSCs have shown some activity in aGvHD and perhaps more convincingly in cGvHD. Nonetheless, a number of hurdles still need to be overcome to make these drugs more effective *in vivo* for GvHD as well as other diseases: we need to better understand the heterogeneity of MSCs due to donor, cell source, subsets, culture conditions, using more extensive and refined methods, which should include more standardized or disease- and function-specific potency assays; we need to identify reliable biomarkers *in vivo*, to predict which patients will be responders, and to more precisely follow the early and late clinical response *in vivo*; we need to investigate and refine best dosing and schedules of administration according to disease type and stage, for examples investigating repeated dosages for GvHD in an early setting.

We believe that MSCs have not yet said their last word and that well conducted studies will bring a more consolidated clinical use of these cells in the future.

## Author Contributions

Both authors have written, discussed, and structured the manuscript. All authors contributed to the article and approved the submitted version.

## Funding

This work was in part funded by AIRC (AIRC 5 × 1000 grant ISM), by the Lombardy Region project Plagencell CP_10/2018), by the Agenzia Italiana del Farmaco (AIFA, Rome, Italy; project MESNEPH) and by the European Commission (Horizon 2020 project NEPHSTROM grant agreement no. 634086). The materials presented and views expressed here are the responsibility of the authors only. The EU Commission takes no responsibility for any use made of the information set out.

## Conflict of Interest

The authors declare that the research was conducted in the absence of any commercial or financial relationships that could be construed as a potential conflict of interest.
